# Differences in associations between attention‐deficit hyperactivity disorder symptoms and physical activity across childhood and adolescence and among males and females in a representative UK birth cohort

**DOI:** 10.1002/jcv2.70146

**Published:** 2026-07-12

**Authors:** Amandine Sénéquier, Stamatina Iliodromiti, Jessica Agnew‐Blais

**Affiliations:** ^1^ Department of Psychology Centre for Brain and Behaviour Queen Mary University of London London UK; ^2^ Women's Health Research Unit Centre for Public Health & Policy Queen Mary University of London London UK

**Keywords:** attention‐deficit hyperactivity disorder, children and adolescents, physical activity, sex differences

## Abstract

**Background:**

Children and adolescents with attention‐deficit hyperactivity disorder (ADHD) exhibit higher physical activity (PA) levels than peers without ADHD. It remains unclear how this association evolves across developmental stages and differs between sexes. This study explores associations between ADHD symptoms and PA levels in childhood and adolescence, focusing on sex differences.

**Methods:**

Data from the Millennium Cohort Study at ages 7 (*n* = 14,043), 11 (*n* = 13,469), 14 (*n* = 11,872) and 17 (*n* = 10,757) were included. Moderate‐to‐vigorous PA (MVPA) was objectively measured using accelerometers at ages 7 and 14, and PA by parent‐ and self‐reporting at ages 7, 11 and 14. ADHD symptoms were assessed by the ADHD subscale of the Strengths and Difficulties Questionnaire (SDQ) at all ages. Multiple linear regressions and mixed‐effects regression models estimated associations between PA and SDQ ADHD scores adjusting for confounders.

**Results:**

MVPA was associated with higher SDQ ADHD scores at age 7 (*b* = 0.009, *p* < 0.001) and, to a lesser extent, age 14 (*b* = 0.004, *p* = 0.004). MVPA and SDQ ADHD scores were associated among males at ages 7 (*b* = 0.008, *p* = 0.001) and 14 (*b* = 0.004, *p* = 0.007), but among girls at age 7 only (*b* = 0.011, *p* < 0.001). Age 7 MVPA was associated with higher ADHD symptoms at ages 11 (*b* = 0.004, *p* = 0.003) and 17 (*b* = 0.004, *p* = 0.031), however age 14 MVPA was not associated with ADHD symptoms at age 17. Higher reported PA levels at ages 7, 11 and 14 were associated with lower SDQ ADHD scores.

**Conclusion:**

Young people with higher objectively‐measured PA levels exhibited higher ADHD symptoms, although this association was stronger in males. By adolescence, PA and SDQ ADHD scores were less strongly associated, and no longer significantly associated among adolescent females. Subjectively‐measured PA showed the inverse association with ADHD symptoms, emphasising the importance of mode of PA measurement.

## Key Points

**What's known?**

- Young people with attention‐deficit hyperactivity disorder (ADHD) tend to be more physically active than their peers without ADHD.
- Accelerometer‐measured physical activity (PA) can predict ADHD symptoms years later.

**What's new?**

- Higher objectively‐measured PA is associated with greater ADHD symptoms in real‐world settings, with stronger associations in childhood than adolescence.
- Associations between PA and ADHD symptoms appear stronger in males than in females, especially in adolescence.

**What's relevant?**

- Findings highlight the importance of considering developmental stage and sex, as well as the link between PA and ADHD, in the design of PA interventions for ADHD, with implications for researchers, clinicians, educators and policy makers.

## INTRODUCTION

Attention‐deficit hyperactivity disorder is a common neurodevelopmental condition characterised by impairing levels of hyperactivity, impulsivity or inattention that affects approximately 7% of children and adolescents (Thomas et al., 2015) and 2.5%–5% of adults globally (Simon et al., 2009). Sex differences in ADHD diagnosis prevalence are well documented, especially in childhood, with male‐to‐female ratios ranging from 2:1 in community or population studies, to 4:1 in clinical settings (Faraone et al., 2015). While the sex ratio is strongly biased towards males in childhood, it is more equal across sexes in adulthood (Hinshaw et al., 2022). This may partly reflect under‐recognition and underdiagnosis of ADHD in females during childhood due to sex differences in symptom presentation (Quinn & Madhoo, 2014). Adolescence may therefore be an important period to investigate sex and ADHD.

Adolescence is characterised by rapid physical and emotional development, and is a key period for the formation of lasting health‐related behaviours that contribute to current and future health (Inchley et al., 2020). An important health behaviour is physical activity (PA), defined as any bodily movement which engages skeletal muscles and requires energy expenditure, including activities relating to transport, work or leisure time (World Health Organization, 2024). The WHO recommends children and adolescents engage in an average of at least 60‐min per day of moderate‐to‐vigorous PA (MVPA) accumulated across the week (World Health Organization, 2024). Exercise is a subcategory of PA, which is intentionally planned, structured and repetitive, and aims to improve or maintain physical fitness (Caspersen et al., 1985). Physical activity has many benefits, such as improved cardiorespiratory, metabolic and musculoskeletal health, and better cognitive function, mental wellbeing and overall quality of life (World Health Organization, 2024). Despite these well‐documented benefits, 81% of adolescents aged 11–17 do not meet WHO PA guidelines, with fewer girls than boys meeting recommendations (World Health Organization, 2024).

Children with ADHD are, on average, more physically active during the day and night compared to their peers without ADHD (Alderson et al., 2012; Cohen‐Zion & Ancoli‐Israel, 2004; Porrino et al., 1983). Several studies examining PA levels using actigraphy in laboratory settings support this association: for example, Rapport et al. (2009) found that children with ADHD moved significantly more than those without during working memory tasks, and Wood et al. (2009), reported that children with ADHD exhibited an increased intensity and variability of movement during laboratory testing. Fewer studies have examined the associations between ADHD and PA in ecologically valid settings (e.g., children wearing activity monitors in daily life). Brandt et al. (2021), using the data from the Millennium Cohort Study (MCS), found that less sedentary behaviour at age 7 predicted ADHD symptoms at age 14. Ahn et al. (2018) found that more time spent in MVPA at age 7 was associated with greater hyperactivity scores at age 11. Another common method for collecting information on PA are self‐report questionnaires, which may offer more context to PA in children's everyday lives, however their validity against device‐based PA measures remains poor‐to‐moderate (Tcymbal et al., 2024).

Despite previous findings that children with ADHD are more active than children without ADHD, there is limited research examining this association during adolescence, or considering sex differences. ADHD symptoms change across development: hyperactivity‐impulsivity symptoms tend to decline, while inattention symptoms are more stable (Willcutt, 2012)—the potential effect of this developmental change on the association with PA has not been investigated. Additionally, females tend to exhibit more symptoms of inattention versus hyperactivity‐impulsivity, and sex differences in the relationship between ADHD and PA from childhood into adolescence have not been assessed.

The aim of this study was to explore the relationship between ADHD symptoms and objectively‐measured PA spanning from childhood into adolescence, focusing on changes across development, potential differences between sexes and variation by ADHD symptom domain (hyperactivity‐impulsivity vs. inattention). We also examined how subjective PA reports aligned with objectively‐measured PA and were associated to ADHD symptoms across development for both sexes.

## METHODS

### Sample

Participants were members of the MCS, a longitudinal study tracking the lives of approximately 19,000 individuals born between 2000 and 2002 in the UK (Centre for Longitudinal Studies, 2023). The MCS collects data on physical, cognitive, socio‐emotional and behavioural development (Centre for Longitudinal Studies, 2023). Cohort members were recruited via random sampling using all listed parents on the UK Child Benefit registers (Plewis et al., 2007). The MCS employed a geographically clustered and disproportionally stratified design to oversample individuals from Scotland, Wales and Northern Ireland, as well as those from disadvantaged backgrounds and areas with higher concentrations of families of ethnic minorities (Plewis et al., 2007). This study used data from MCS Sweep 4 (MCS4) (age 7, *n* = 14,043), Sweep 5 (MCS5) (age 11, *n* = 13,469), Sweep 6 (MCS6) (age 14, *n* = 11,872) and Sweep 7 (MCS7) (age 17, *n* = 10,757) (see Supporting Information S1: Appendix S1, Figure S1 for sample details). Parents and guardians provided informed written consent and cohort members provided assent and consent as they aged (Chaplin Gray et al., 2010; *Millennium Cohort Study Sixth Sweep (MCS6): Technical report* 2017). Ethical approval for all sweeps was obtained by the Centre for Longitudinal Studies (CLS) (Chaplin Gray et al., 2010; Gallop et al., 2013; *Millennium Cohort Study Seventh Sweep (MCS7): Technical report*, 2019; *Millennium Cohort Study Sixth Sweep (MCS6): Technical report* 2017).

### Measures

#### ADHD symptom measure

To assess ADHD symptoms, caregivers completed the Strengths and Difficulties Questionnaire (SDQ), a brief behavioural screening questionnaire for 4‐17‐year‐olds (Goodman, 2001), and cohort members completed the SDQ themselves at age 17 (MCS7). The SDQ ADHD subscale assessed hyperactivity‐impulsivity (“restless, overactive, cannot stay still for long”, “constantly fidgeting or squirming” and “thinks things out before acting”) and inattention (“easily distracted, concentration wanders” and “sees tasks through to the end, good attention span”) (Hansen et al., 2014). The total SDQ ADHD subscale score was calculated (ranging from 0 to 10), along with individual dimensional scores for hyperactivity‐impulsivity (0–6) and inattention (0–4) by summing respective items. The items of the SDQ ADHD subscale have acceptable internal consistency (average Cronbach's *α*  = 0.77–0.87) (Brandt et al., 2021; Burley et al., 2022). The SDQ ADHD subscale has been widely validated as a screening instrument for ADHD in both clinical and community samples (Hall et al., 2019; Riglin et al., 2016) and correlates well with clinician‐rated ADHD symptoms (Mathai et al., 2003). A clinical cut‐off score of ≥7 was applied to characterise low/moderate (ranging from 0 to 6) and high ADHD symptoms (ranging from 7 to 10) groups, with a score of 6 being considered a borderline score (Riglin et al., 2016).

#### Physical activity

##### Objectively‐measured MVPA

Accelerometer data were collected at ages 7 and 14. At age 7, all cohort members were invited to participate in the accelerometer study (Griffiths et al., 2013); at age 14, 81% of cohort members living in England and all those living in Scotland, Wales and Northern Ireland were invited (Fitzsimons et al., 2020). At age 7, PA was recorded using the Actigraph GT1M uni‐axial accelerometer (Actigraph, Pensacola, Florida) in counts and the sampling epoch was set at 15 s (Griffiths et al., 2013). Cohort members wore the accelerometer on the right hip for seven consecutive days, excluding aquatic activities (Griffiths et al., 2013). At age 14, the GENEActiv Original triaxial accelerometer recorded PA based on the mean acceleration using the Euclidean Norm Minus One and the sampling epoch was set at 5 s (Fitzsimons et al., 2020). Cohort members wore the accelerometer on their wrist on one random weekday and one weekend day (Fitzsimons et al., 2020). Non‐wear time was considered any consecutive period of zero‐counts lasting at least 20 min at age 7 (Griffiths et al., 2013) and any consecutive period when the standard deviation of the raw signal from at least two of the three axes was below 13 milli‐gravitational units (mg) and the value range was less than 50 mg for at least 15 min at age 14 (Heywood, 2018). Accelerometer data were considered reliable with a minimum wear period of two days of at least 10 h each at age 7 (Griffiths et al., 2013), while a minimum wear period of one day with at least 10 valid hours was considered reliable at age 14 (*n* = 4,113, 86.59% of cohort members had data for both days at age 14) (Heywood, 2018). Summary variables for mean daily minutes spent in MVPA were derived at ages 7 (2241‐11,714 counts/minute) (Griffiths et al., 2013) and 14 (ENMO > 100 mg for >80%/minute) (Heywood, 2018). Both accelerometers have demonstrated acceptable reliability and validity (Phillips et al., 2013; Plasqui & Westerterp, 2007; Trost et al., 2006).

##### Parent‐ and self‐reported PA

Parent‐reported PA was assessed via cohort members' main caregivers at ages 7 and 11 using the question, “How many days a week does [cohort member] usually go to a club or class to do sport or any other PA like swimming, gymnastics, football, dancing, etc.?”, with possible answers ranging from, “Not at all” to, “Five or more days a week” (*Millennium Cohort Study Fifth Sweep (MCS5): Age 11 survey, Household and main/partner questionnaire*, 2013; *Millennium Cohort Study Sweep 4: Questionnaire documentation*, 2009). This was self‐reported by the cohort member at age 11 using the question “How often do you play sports or active games inside or outside, not at school?”, with possible answers ranging from, “Never” to, “Most days” (*Child of the New Century, Question booklet*, 2013). It was also self‐reported at age 14, using the question, “On how many days in the last week did you do a total of at least an hour of moderate to vigorous PA?”, with possible answers ranging from, “Not at all” to, “Every day” (*Millennium Cohort Study Sweep 6: Young person questionnaire*, 2015).

### Statistical analysis

Only the first sibling from each multiple birth family was included to avoid potential confounding effects related to family membership (Reed et al., 2024). Descriptive characteristics of the sample were survey‐weighted means and standard deviations for continuous variables, and count and frequencies for categorical variables. Independent *t*‐tests and χ^2^ tests were conducted to measure differences between sexes. The Kendall's coefficient of rank correlation tau‐sub‐b (τb) was calculated to assess correlation between objectively and ordinally assessed parent‐ and self‐reported PA at ages 7 and 14 (Khamis, 2008). Multiple linear regression models estimated associations between SDQ ADHD scores and objective PA at ages 7 and 14 and subjective PA at ages 7, 11 and 14. Mixed effect models with random intercepts for participant, and an interaction term between PA and wave, were fitted to investigate change in associations between SDQ ADHD score and accelerometer‐measured PA between age 7 and age 14. To assess whether this differed by sex, a three‐way interaction between MVPA, wave and sex was included in the mixed effect models and a Wald test was performed. Multiple linear regression models assessed whether objectively‐measured PA at age 7 predicted SDQ ADHD scores at ages 11, 14 and 17, and whether age 14 PA predicted SDQ scores at age 17. In all models, we examined the SDQ ADHD score, along with scores for hyperactivity‐impulsivity and inattention. All models were stratified by sex. Analyses were adjusted for potential confounders, including sex, age, birth month, body mass index (BMI: derived as weight (kg)/height (m)^2^ and recorded only when both measures were valid), ethnicity (the six category census), household income (Organisation for Economic Co‐operation and Development equivalised income quintiles), parental education (highest National Vocational Qualifications equivalent academic qualification) and season of accelerometer wear (seasons were assigned by solstice/equinox dates for MCS4 and by accelerometer wear month for MCS6) (Ahn et al., 2018; Brandt et al., 2021). Benjamini‐Hochberg false discovery rate (FDR) correction (*q* = 0.05) was applied separately to three families of tests: (1) cross‐sectional objective MVPA; (2) cross‐sectional subjective PA; and (3) longitudinal regression models predicting SDQ ADHD scores from previously measured MVPA (Benjamini & Hochberg, 1995). For categorical subjective PA variables, an omnibus *F*‐test *p*‐value was used. An inverse probability weighting approach was used to account for missing data, the sampling design of the MCS and the smaller sample size of the accelerometer data, and applied in all analyses, except for the correlations between objectively and subjectively‐measured PA (Ketende & Jones, 2011). Details on weight construction are provided in Supporting Information S1: Appendix S2, while data availability and missingness for accelerometer‐measured MVPA and SDQ ADHD scores are shown in Supporting Information S1: Appendix S1, Figure S1, with valid data for 6741 participants at age 7 and 4652 at age 14. Analyses were conducted using Stata V.19 (StataCorp, 2023).

## RESULTS

### Sample characteristics

Table 1 presents descriptive characteristics at ages 7 and 14 by sex. Mean SDQ ADHD score declined from age 7 to 14 and was significantly higher for males than females. Slightly over 10% of participants scored above the clinically meaningful threshold of 7 on the SDQ ADHD scale. At age 7, 50.70% of children met PA recommendations (≥60 min MVPA/day), decreasing to 41.24% by age 14. Mean daily number of MVPA minutes declined with age. Males engaged in significantly more MVPA than females and were more likely to meet PA recommendations at both ages (*p* < 0.001).

**TABLE 1:** Descriptive characteristic of the sample at age 7 and age 14.

	Age 7 (MCS4)			Age 14 (MCS6)		
	Combined *n* = 6502	Females *n* = 3194	Males *n* = 3308	Combined *n* = 4670	Females *n* = 2264	Males *n* = 2406
SDQ ADHD score^a^	3.30 (2.44)	2.86 (2.33)	3.73 (2.47)***	3.05 (2.47)	2.62 (2.33)	3.45 (2.52)***
SDQ hyperactivity score (ranging from 0 to 6)^a^	1.89 (1.54)	1.65 (1.48)	2.13 (1.57)***	1.67 (1.52)	1.47 (1.42)	1.86 (1.57)***
SDQ inattention score (ranging from 0 to 4)^a^	1.38 (1.19)	1.19 (1.15)	1.58 (1.20)***	1.38 (1.24)	1.16 (1.21)	1.60 (1.23)***
SDQ categories^b^						
<7	5618 (88.25)	2874 (91.97)	2743 (84.66)	4101 (89.41)	2082 (93.46)	2019 (85.58)
≥7	748 (11.75)	251 (8.03)	497 (15.34)	486 (10.59)	146 (6.54)	340 (14.42)
MVPA (mins)^a^	63.16 (22.46)	56.23 (19.56)	69.88 (22.89)***	60.69 (42.85)	52.47 (35.66)	68.41 (46.75)***
Age at measurement (years)^a^	7.14 (0.35)	7.14 (0.35)	7.15 (0.35)	13.78 (0.45)	13.77 (0.45)	13.79 (0.44)
BMI (kg/m^2^)^a^	16.60 (2.39)	16.63 (2.43)	16.57 (2.35)	21.50 (4.22)	22.20 (4.50)	20.87 (3.85)***

*Note*: Using accelerometer weight MCS4 for age 7 descriptives, accelerometer weight for MCS6 for age 14 descriptives.

Abbreviations: ADHD, Attention‐deficit hyperactivity disorder; BMI, Body Mass Index; MCS, Millennium Cohort Study; MVPA, Moderate‐to‐vigorous physical activity; SD, Standard Deviation; SDQ, Strengths and Difficulties Questionnaire.

^a^ Continuous variables presented as mean (SD).

^b^ Categorical variables presented as count (%).

Significant differences between males and females: *p < 0.05, **p < 0.01, ***p < 0.001.

### ADHD symptoms and accelerometer‐measured MVPA

#### Cross‐sectional associations between ADHD symptoms and objectively‐measured MVPA

At age 7, higher MVPA was significantly positively associated with higher SDQ ADHD scores in the total sample and among females and males separately (Table 2). At age 14, higher MVPA was associated with higher SDQ ADHD scores overall and among males, but not among females. Results were similar for hyperactivity symptoms, where at age 7 higher MVPA was associated with higher SDQ ADHD scores among females and males, and while this remained significant at age 14 overall and for males, it was no longer significant among females. For inattention symptoms, at age 7 there were no associations with MVPA; at age 14, results were similar to results for ADHD symptoms overall, with significant associations in the overall sample and among males, but not among females. All *p*‐values remained significant after multiple testing correction (5% FDR).

**TABLE 2:** Associations between MVPA and SDQ ADHD, hyperactivity and inattention scores at ages 7 and 14 in females and males.

		Total		Hyperactivity		Inattention	
		b	95% CI	b	95% CI	b	95% CI
Age 7 (MCS4) *n* = 6340							
Combined	MVPA	0.009***	0.005–0.012	0.007***	0.005–0.010	0.001	−0.0002–0.003
Females	MVPA	0.011***	0.005–0.016	0.008***	0.005–0.012	0.002	−0.0002–0.005
Males	MVPA	0.008**	0.003–0.013	0.007***	0.004–0.010	0.001	−0.001–0.003
Age 14 (MCS6) *n* = 4375							
Combined	MVPA	0.004**	0.001–0.006	0.002**	0.0006–0.004	0.001*	0.0002–0.003
Females	MVPA	0.003	−0.001–0.007	0.002	−0.0005–0.004	0.001	−0.001–0.003
Males	MVPA	0.004*	0.001–0.007	0.002*	0.0003–0.004	0.002*	0.0002–0.003

*Note*: Using MCS4 accelerometer weight for age 7, using MCS6 accelerometer weight for age 14. All *p*‐values remained significant after multiple testing correction (5% false discovery rate [FDR]).

Abbreviations: ADHD, Attention‐deficit hyperactivity disorder; CI, Confidence interval; b, Regression coefficient; SDQ, Strengths and Difficulties Questionnaire.

*p < 0.05, **p < 0.01, ***p < 0.001.

#### Comparing the association between ADHD symptoms and objectively‐measured MVPA at ages 7 and 14

**FIGURE 1:**
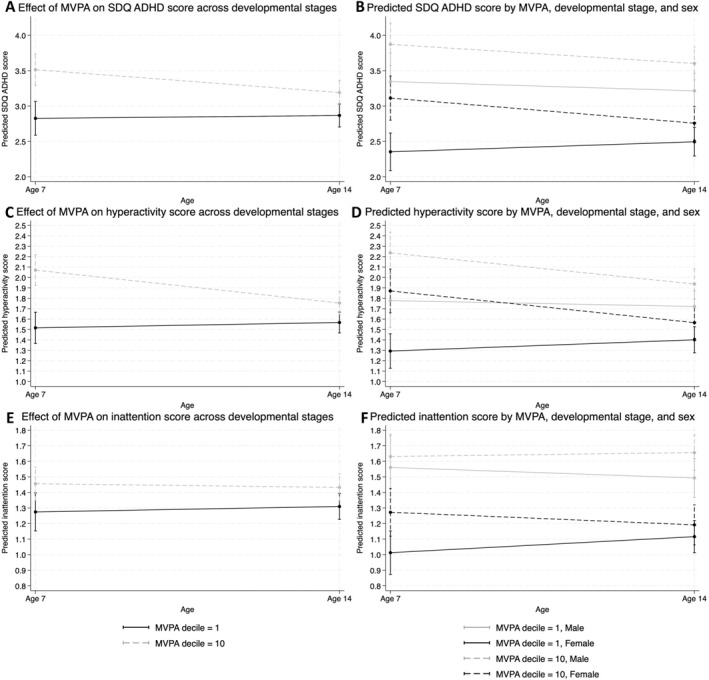
Results from the mixed effect multiple linear regression analyses regarding SDQ ADHD score, hyperactivity only and inattention only symptoms across waves and in females and males. MVPA was grouped into deciles for clearer visual representation. ADHD, attention‐deficit hyperactivity disorder.

Mixed effect multiple linear regression analyses investigating whether the association between MVPA and ADHD symptoms differed from ages 7 to 14 in the overall sample did not reach statistical significance (Figure 1, panel A; full results in Supporting Information S1: Appendix S3, Table S1). Additionally, a three‐way interaction between age, sex and MVPA was not significant, suggesting the MVPA and ADHD symptoms association over time does not significantly differ between males and females (Figure 1B; full results in Supporting Information S1: Appendix S3, Table S2). Looking separately at symptom domains, we found the association between hyperactivity symptoms and MVPA was significantly weaker at age 14 than age 7 (with no difference by sex) (Figure 1, panels C and D; full results in Supporting Information S1: Appendix S3, Tables S1 and S2). The association between inattention and MVPA did not differ by age or by sex (Figure 1, panels E and F; full results in Supporting Information S1: Appendix S3, Tables S1 and S2).

#### Predicting ADHD symptoms at age 11, 14 and 17 using previous objectively‐measured MVPA

MVPA at age 7 was significantly associated with higher SDQ ADHD scores at age 11, over and above ADHD symptoms measured at age 7, but this effect was significant only in the overall sample and among males (Table 3). However, looking to older ages (14 and 17), MPVA at age 7 was significantly associated with higher SDQ ADHD scores only among males; amongst females and in the overall sample this did not reach statistical significance. However, none of these associations survived FDR correction (*q* = 0.05) and should be interpreted with caution. MVPA at age 14 was not significantly associated with SDQ ADHD scores at age 17 in any group.

**TABLE 3:** Regression models predicting SDQ ADHD score separately at ages 11, 14 and 17.

		Age 11 SDQ ADHD score		Age 14 SDQ ADHD score		Age 17 SDQ ADHD score	
		*n* = 5294		*n* = 4699		Age 7, *n* = 2449 Age 14, *n* = 2111	
		b	95% CI	b	95% CI	b	95% CI
Combined	Age 7 MVPA	0.004**	0.001–0.007	0.004	−0.0002–0.009	0.0004	−0.003–0.004
Females	Age 7 MVPA	0.002	−0.002–0.006	−0.001	−0.007–0.004	0.003	−0.002–0.008
Males	Age 7 MVPA	0.006**	0.002–0.01	0.008*	0.002–0.01	0.006*	0.0004–0.01
Combined	Age 14 MVPA					0.0006	−0.001–0.002
Females	Age 14 MVPA					−0.0005	−0.003 to 0.002
Males	Age 14 MVPA					0.001	−0.001–0.003

*Note*: Using MCS4 accelerometer weight for age 7, using MCS6 accelerometer weight for age 14. No *p*‐values were significant after multiple testing correction (5% false discovery rate [FDR]).

Abbreviations: ADHD, Attention‐deficit hyperactivity disorder; CI, Confidence interval; b, Regression coefficient; SDQ, Strengths and Difficulties Questionnaire.

*p < 0.05, **p < 0.01, ***p < 0.001.

#### Predicting self‐reported ADHD symptoms at age 17 using previous objectively‐measured MVPA

At age 17, ADHD SDQ score was also assessed via self‐reporting by the cohort member. MVPA at age 7 was significantly associated with higher self‐reported SDQ ADHD score at age 17 in the overall sample (*b* = 0.004, *p* < 0.05) and among males (*b* = 0.006, *p* < 0.05), over and above ADHD symptoms measured contemporaneously (Supporting Information S1: Appendix S4, Table S3). However, neither association survived FDR correction (*q* = 0.05) and should be interpreted with caution. Similar to parent‐reported SDQ ADHD score, MVPA at age 14 was not significantly associated with self‐reported SDQ ADHD scores at age 17 in any group.

### Parent‐ and self‐reported physical activity and ADHD symptoms

At age 7, parents reported 33.16% of children exercised less than once a week, with 7.43% exercising ≥4 days/week. At age 11, these figures were 27.41% and 15.84%, respectively. At age 14, about 5% of adolescents self‐reported never engaging in ≥60 min MVPA/day, while 19.03% reported doing so daily. Females were consistently more likely to report never exercising or not engaging in ≥60 min MVPA/day (*p* < 0.05). They were also less likely to report exercising ≥4 days/week or engaging in ≥60 min MVPA/day every day (*p* < 0.05).

#### Correlations between reported and objectively‐measured physical activity

**FIGURE 2:**
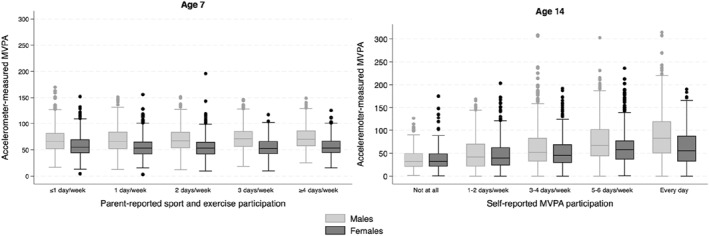
Distribution of accelerometer‐measured MVPA by reported physical activity (PA) by age and sex. MVPA, Moderate‐to‐vigorous PA.

Accelerometer‐measured PA was significantly positively correlated with both parent‐ and self‐reported sport/exercise participation, with a stronger association with age 14 self‐reported MVPA than age 7 parent‐reported sport/exercise participation (Figure 2, age 7: τb = 0.031, *p* = <0.001; age 14: τb = 0.200, *p* < 0.001). Correlations were stronger in males for both parent‐reported sport/exercise participation and self‐reported MVPA (age 7: τb = 0.055, *p* < 0.001; age 14: τb = 0.214, *p* < 0.001) than in females (age 7: τb = −0.007, *p* = 0.587; age 14: τb = 0.152, *p* < 0.001). Although associations between reported and accelerometer‐measured PA were statistically significant, the effect sizes were small.

#### Associations between reported ADHD symptoms and reported physical activity

Higher levels of parent‐reported sport/exercise participation at ages 7 and 11, self‐reported sport/exercise participation at age 11, and self‐reported MVPA engagement at age 14 were significantly associated with lower SDQ ADHD (age 7: *b* = −0.23 to −0.42; age 11 parent‐reported: *b* = −0.38 to −0.57; age 11 self‐reported: *b* = −0.71 to −1.08; age 14: *b* = −0.43 to −0.81), hyperactivity and inattention scores. Similar results were observed in females and males (Supporting Information S1: Appendix S5, Tables S4–S7). All *p*‐values remained significant after multiple testing correction (5% FDR). All results of FDR corrected analyses are including in Supporting Information S1: Appendix S6, Tables S8–S10.

## DISCUSSION

Our study found that the associations between objectively‐measured PA and ADHD symptoms were twice as strong in childhood than in adolescence. These associations were significant in both sexes at age 7, but only in males at age 14, highlighting sex differences. The associations between objectively‐measured MVPA and parent‐ and self‐reported PA were statistically significant but of small effect size, and reported PA had the inverse relationship with ADHD symptoms compared with objectively‐measured PA, with higher reported PA associated with lower ADHD symptoms.

### Physical activity more strongly associated with ADHD symptoms in childhood than adolescence

While 15%–70% of children with ADHD continue to display ADHD symptoms in adolescence (Faraone et al., 2006; Langley et al., 2010), the manifestation of ADHD symptoms evolves, with symptoms of hyperactivity tending to decline more rapidly than inattention, which remain more stable (Francx et al., 2015; Willcutt, 2012). Supporting the decrease in the prominence of motor hyperactivity during adolescence, our longitudinal models revealed that the strength of the association between MVPA and hyperactivity symptoms decreases between childhood and adolescence. This may also be explained by an internalisation of hyperactivity symptoms, such that hyperactivity may manifest as more subjective feelings of internal restlessness, or shift from more overt behaviours (e.g., excessive running or leaving seats) in childhood to more subtle behaviours such as fidgeting in adolescence (Son et al., 2024; Zalsman & Shilton, 2016). Our finding of an emergence of an association between PA and inattention symptoms during adolescence compared with childhood, may stem from the increasing importance of inattention symptoms in ADHD during this period. Overall MVPA declines in the transition between childhood and adolescence (Okazaki et al., 2022), and adolescents have less opportunity to participate in child‐driven spontaneous activities that are relatively unstructured and undertaken freely such as active play, which may contribute to the change in association between PA and ADHD symptoms into adolescence.

### Sex differences in the association between physical activity and ADHD symptoms

While girls with higher levels of PA showed higher ADHD symptoms in childhood, there were no significant associations between MVPA and ADHD symptoms in females during adolescence (while differences remained for boys), highlighting sex differences in associations between PA and ADHD symptoms from childhood to adolescence. Type of ADHD symptom may underpin some of these differences, as PA was more strongly associated with hyperactivity symptoms, which are also less frequent among girls (Franke et al., 2018). Additionally, it is possible that females may be more attentive to social norms and expectations and more likely to mask their ADHD symptoms, perhaps rendering associations with more overt PA, like climbing and running around, less noticeable in adolescence.

We also observed a decrease in PA levels from childhood to adolescence, and females spent less time in MVPA compared to males. Sex differences in biological maturation may influence PA participation: maturing females experience physical changes such as breast development, hip widening and increased fat deposition, which may reduce PA participation (Bacil et al., 2015). In contrast, males gain height, body weight and lean mass, perhaps encouraging PA participation (Bacil et al., 2015; Erlandson et al., 2011). Further studies are necessary to investigate the link between PA and pubertal stages, especially among adolescents with ADHD.

### Childhood MVPA is associated with higher ADHD symptoms at older ages

We found that MVPA was associated with higher SDQ ADHD scores cross‐sectionally, as well as longitudinally over and above ADHD symptoms measured contemporaneously. However, it should be noted that while all cross‐sectional associations survived FDR correction, none of the longitudinal associations did, suggesting that these findings are less robust than our cross‐sectional findings and should be replicated in future studies before conclusions can be drawn. A previous study in the same cohort found that lower levels of sedentary activity at age 7 predicted ADHD symptoms at age 14, but did not find any significant associations with light, moderate or vigorous PA levels and later ADHD symptoms (Brandt et al., 2021). This difference may arise from our focus on MVPA, which may be more strongly associated with ADHD symptoms than the broader range of light, moderate and vigorous. These differences between studies highlight how varying measurement approaches, such as intensity‐based categorisation or confounding factors used can influence our interpretation of the effect of PA on ADHD symptoms. Additionally, these associations may reflect residual confounding and potential reverse causation, whereby higher ADHD symptoms lead to increased MVPA.

### Different associations between ADHD symptoms and parent‐ and self‐reported physical activity versus objectively‐measured MVPA

The relationship between ADHD symptoms and PA appears to differ based on the measurement method: while accelerometer‐measured MVPA was positively associated with ADHD symptoms, parent and self‐reported PA was negatively associated with ADHD symptoms. Consistent with this finding, a Swedish cohort study found that greater levels of self‐reported PA in adolescence was significantly associated with lower levels of ADHD symptoms in early adulthood, adjusting for shared genetic and shared environmental factors within monozygotic twin pairs, ADHD symptoms and BMI at baseline (Rommel et al., 2015). Actigraphy measures all PA movement, including hyperactive‐type behaviours (e.g., running around at break time) which are characteristics of ADHD, whereas reported PA is more likely to reflect engagement in structured physical activities. A systematic review commented on the usefulness of accelerometer‐based devices in helping to differentiate between individuals with ADHD and individuals without ADHD and evaluating the effects of medication, suggesting objectively‐measured PA may have stronger associations with ADHD symptoms (Hall et al., 2016).

### Physical activity, exercise and ADHD symptoms

MVPA holds a multifaceted position in its relationship to ADHD symptoms. Importantly, accelerometer‐based measures capture all movement, including activity reflecting hyperactive symptoms. Thus, higher levels of MVPA may reflect behavioural manifestations of ADHD, rather than beneficial PA per se. On one hand, increased levels of MVPA (which can include exercise) may therefore be a potential marker for higher levels of ADHD symptoms, perhaps even distinguishing among children with and without ADHD (Basic et al., 2024). On the other hand, multiple meta‐analyses report that PA and exercise interventions could effectively improve attention, motor skills, executive function, and emotional and behavioural problems in children and adolescents with ADHD (Sun et al., 2022; Xie et al., 2021). Thus, PA may differ between children with higher versus lower ADHD symptoms, while, on a within‐individual level, increases in PA, especially through structured exercise interventions, may be associated with improvements in ADHD symptoms within children. Accordingly, MVPA may simultaneously act as a marker of ADHD symptoms and as a strategy individuals may employ to help manage those symptoms.

There are several strengths of the current study, including investigating both childhood and the adolescent period, as well as sex differences. Additionally, PA was objectively measured via accelerometers, as well as via subjective PA measures, providing a comprehensive assessment of PA. However, results should be interpreted in the context of several limitations. Although several associations were statistically significant, effect sizes were very small and may have limited clinical relevance at the individual level. However, small effects may still be relevant at the population level. Furthermore, several nominally significant longitudinal associations did not survive FDR correction for multiple comparisons and should therefore be interpreted with caution. Accelerometer‐based PA measures do not capture the context or characteristics of PA, which may be important for understanding its association with ADHD, for example they cannot distinguish whether the activity is structured (e.g., participation in a sports club) or unstructured (e.g., leaving one's seat in class). They may also not capture all manifestations of hyperactivity, such as fidgeting or other more subtle repetitive movements. Additionally, there were differences in the accelerometers used at ages 7 and 14—including variations in placement, measurement duration, metrics and sampling epochs—which may limit their direct comparability across developmental stages (Migueles et al., 2017), as well as potentially affecting estimates of PA levels, the interpretation of change and may have contributed to the attenuation of associations observed over time. Another potential limitation is non‐response and non‐compliance in PA monitoring; we aimed to mitigate this potential selection bias with an inverse probability weighting approach.

## CONCLUSION

These findings suggest that the relationship between objectively‐measured PA and ADHD symptoms varies by age and sex. Children who engaged in more MVPA had more ADHD symptoms, both cross‐sectionally and prospectively, with childhood MVPA predicting later ADHD symptoms. In adolescence, higher levels of MVPA were still associated with more ADHD symptoms, but the strength of this relationship was weaker and limited to males. The associations between MVPA and ADHD symptoms were stronger in males than in females, possibly reflecting the predominance of inattention symptoms among females. Overall, these findings highlight the importance of both developmental stage and sex in understanding the link between PA and ADHD symptoms.

## AUTHOR CONTRIBUTIONS

**Amandine Sénéquier**: Conceptualisation; data curation; writing—original draft preparation; writing—review and editing. **Stamatina Iliodromiti**: Conceptualisation; writing—review and editing. **Jessica Agnew‐Blais**: Conceptualisation; statistical analysis; supervision; writing—review and editing.

## CONFLICT OF INTEREST STATEMENT

The authors declare no conflicts of interest.

## ETHICAL CONSIDERATIONS

This study used data from the MCS which were accessed through the publicly available UK Data Service. The NHS Research Ethics Committee provided ethical approval for all sweeps, including approvals from Yorkshire MREC (ref: 07/MRE03/32), Yorkshire and The Humber—Leeds East (ref: 11/YH/0203), London—Central MREC (13/LO/1786) and North East—York (ref: 17/NE/0341). Consent obtained from parent(s)/guardian(s) and the young people where appropriate. As this analysis involved secondary use of fully anonymised, publicly available data, no additional ethical approval or separate date of approval was required.

## Supporting information

Supporting Information S1

## Data Availability

The Millennium Cohort Study data, including the datasets used in this study, are accessible to bona fide researchers through the UK Data Service at the University of Essex (https://beta.ukdataservice.ac.uk/datacatalogue/series/series?id=2000031).
