# Supplementary material for: Differences in associations between attention‐deficit hyperactivity disorder symptoms and physical activity across childhood and adolescence and among males and females in a representative UK birth cohort
Source: JCPP Adv. 2026 Jul 12:e70146. Online ahead of print. doi: 10.1002/jcv2.70146 (PMC13357859; doi:10.1002/jcv2.70146)
Supplement: Supplementary file 1 — Supporting Information S1 [file JCV2-9999-e70146-s001.docx]

# Differences in associations between attention-deficit hyperactivity disorder (ADHD) symptoms and physical activity across childhood and adolescence and among males and females in a representative UK birth cohort

## Supporting Information

### Appendix S1 – Description of the sample

### Figure S1. Flow diagram of the MCS cohort participation, accelerometer distribution and return and data availability across sweeps.

### Appendix S2 – Inverse probability weighting approach

### Appendix S3 – Longitudinal associations between ADHD symptoms and accelerometer-measured MVPA

### Table S1. Weighted results from the mixed effects multiple linear regression models used to investigate whether the associations between ADHD symptoms and PA differs between childhood (age 7) and adolescence (age 14).

### Table S2. Weighted results from the mixed effects multiple linear regression models used to investigate whether the associations between ADHD symptoms and PA between females and males both at age 7 and age 14.

### Appendix S4 – Predicting self-reported ADHD symptoms at age 17 using previous accelerometer-measured MVPA

### Table S3. Regression models predicting self-reported SDQ ADHD score separately at age 17.

### Appendix S5 – Associations between reported ADHD symptoms and reported physical activity

### Table S4. Weighted results from the multiple linear regression analyses regarding age 7 (MCS4) SDQ ADHD, hyperactivity symptoms only and inattention symptoms only using parent-reported subjective physical activity levels: regression coefficients and 95% CIs.

### Table S5. Weighted results from the multiple linear regression analyses regarding age 11 (MCS5) SDQ ADHD, hyperactivity symptoms only and inattention symptoms only using parent-reported subjective physical activity levels: regression coefficients and 95% CIs.

### Table S6. Weighted results from the multiple linear regression analyses regarding age 11 (MCS5) SDQ ADHD, hyperactivity symptoms only and inattention symptoms only using self-reported subjective physical activity levels: regression coefficients and 95% CIs.

### Table S7. Weighted results from the multiple linear regression analyses regarding age 14 (MCS6) SDQ ADHD, hyperactivity symptoms only and inattention symptoms only using self-reported subjective physical activity levels: regression coefficients and 95% CIs.

### Appendix S6 – Benjamini Hochberg corrections

### Table S8. Research question 1

### Table S9. Research question 2

### Table S10. Research question 3

### Appendix S1 – Description of the sample


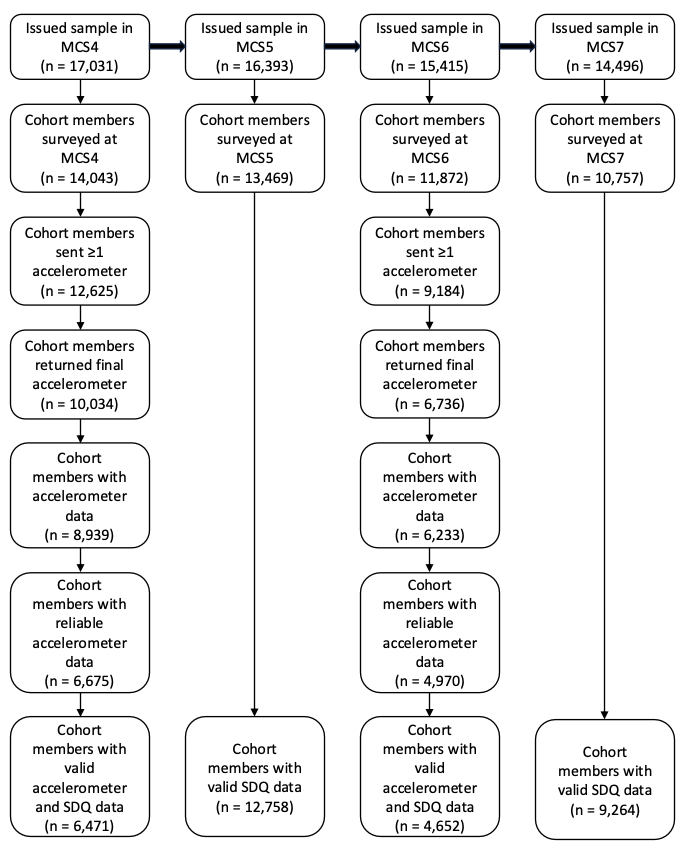


**Figure S1**. Flow diagram of the MCS cohort participation, accelerometer distribution and return and data availability across sweeps. The figure illustrates the extent of missing data in the main variables of interest, including accelerometer data and SDQ scores at each stage.

MCS, Millennium Cohort Study; PA, physical activity; SDQ, Strengths and Difficulties Questionnaire.

###

### Appendix S2 – Inverse probability weighting approach

In the Millennium Cohort Study (MCS), physical activity was measured using accelerometers. In the fourth sweep of the MCS, all cohort members were invited to participate. However, in the sixth sweep, although all cohort members in Scotland, Wales, and Northern Ireland were invited to the accelerometer study, only 81% of those in England were invited (Agalioti-Sgompou et al., 2020). Additionally, there were missing data due to unit non-response and non-compliance to the study protocol. To mitigate potential selection bias from differences in who did and who did not provide accelerometer data, we used an inverse probability weighting approach (Mansournia & Altman, 2016).

In the fourth sweep of MCS, an overall weight variable for whole UK analyses was created for the accelerometer study. Details of the procedure to create this fourth sweep of MCS weight variable are available in the [Technical report on the enhancement of Millennium Cohort Study data with accelerometer-derived measures of physical activity and sedentary behaviour in seven year olds](https://doc.ukdataservice.ac.uk/doc/7238/mrdoc/pdf/mcs4_pa_technical_report.pdf). This weight variable was available in the MCS sweep 4 datasets.

However, no such weight variable was available in the MCS sweep 6 (MCS6) datasets. Therefore, we created the corresponding MCS6 weight variable using a simplified approach to the one described in the Technical report mentioned above. Briefly, probability weights were predicted using a logistic regression model. The predictors in the model included some of the variables used by Plewis et al. (2007) (e.g., UK country, ethnicity, housing tenure), as well as child-level predictors (e.g., sex, obesity status). The estimation accounted for the MCS accelerometer survey-design features including specific country accelerometer distribution. Predicted inverse probabilities were multiplied by MCS6 non-response adjusted sampling weights to give overall weights. These were rescaled to the number of participants available in the dataset.

More precisely, the predictors included in the logistic regression models were: sex (MCS6), age (MCS6), number of days last week spend doing moderate-to-vigorous physical activity (MCS6), ethnicity (MCS6), obesity flag (MCS6), summary of parents/carers in household (MCS6), number of siblings in household (MCS6), OECD equivalised income quintiles UK whole (MCS6), NVQ equivalent of highest academic level across sweeps (MCS6), country at interview (MCS6), cohort member conditions/illnesses limit every day activities (MCS6), housing tenure (MCS6), and ever tried to breastfed (MCS1).

References:

Agalioti-Sgompou, V., Calderwood, L., Gilbert, E., Haselden, L., & Johnson, J. (2020). *Millennium Cohort Study Sixth Survey 2015-2016: user guide (2nd Edition)*.

Griffiths, L., Rich, C., Geraci, M., Sera, F., Cortina-Borja, M., Pouliou, T., et al. (2013). *Technical report on the enhancement of Millennium Cohort Study data with accelerometer-derived measures of physical activity and sedentary behaviour in seven year olds.* London, UK: Centre for Longitudinal Studies, University College London Institute of Education.

Mansournia, M. A., & Altman, D. G. (2016). Inverse probability weighting. *British Medical Journal (Clinical research ed.)*, *352*. <https://doi.org/10.1136/BMJ.I189>

Plewis, I., Calderwood, L., Hawkes, D., Hughes, G., & Joshi, H. (2007). *The Millennium Cohort Study: Technical Report on Sampling (4th Edition)* (1898453624). [www.ioe.ac.uk/bedfordgroup](file:///C:\Users\jessica\Desktop\LabGroupStuff\Amandine\jccpa_resubmission\www.ioe.ac.uk\bedfordgroup)

### Appendix S3 – Longitudinal associations between ADHD symptoms and accelerometer-measured MVPA

Table S1. Weighted results from the mixed effects multiple linear regression models used to investigate whether the associations between ADHD symptoms and PA differs between childhood (age 7) and adolescence (age 14).

|  | SDQ ADHD  n = 4,608 | | | Hyperactivity symptoms only  n = 4,552 | | | Inattention symptoms only  n = 4,583 | | |
| --- | --- | --- | --- | --- | --- | --- | --- | --- | --- |
| Predictors | b | 95% CI | | b | 95% CI | | b | 95% CI | |
| Model 1 | | | | | | | | | |
| MVPA | 0.005*** | 0.002 | 0.007 | 0.002** | 0.0009 | 0.004 | 0.002** | 0.001 | 0.003 |
| Model 2 | | | | | | | | | |
| MVPA | 0.01*** | 0.005 | 0.014 | 0.007*** | 0.004 | 0.01 | 0.004** | 0.001 | 0.006 |
| Wave (Ref: Wave 1 – MCS4)  *Wave 2 – MCS6* | 0.12 | -0.19 | 0.44 | 0.12 | -0.09 | 0.33 | 0.11 | -0.05 | 0.27 |
| Wave##MVPA (Ref: Wave 1 – MCS4)  *Wave 2 – MCS6##MVPA* | -0.006* | -0.011 | -0.0008 | -0.005** | -0.009 | -0.002 | -0.002 | -0.004 | 0.0007 |
| Model 3 | | | | | | | | | |
| MVPA | 0.008** | 0.004 | 0.013 | 0.007*** | 0.003 | 0.01 | 0.002* | 0.0002 | 0.005 |
| Wave (Ref: Wave 1 – MCS4)  *Wave 2 – MCS6* | 0.11 | -0.25 | 0.47 | 0.13 | -0.11 | 0.36 | 0.05 | -0.13 | 0.23 |
| Wave##MVPA (Ref Wave 1 – MCS4)  *Wave2 – MCS6##MVPA* | -0.005 | -0.01 | 0.0004 | -0.005** | -0.008 | -0.001 | -0.0009 | -0.003 | 0.002 |
| Sex (Ref: Male)  *Female* | -0.79*** | -0.98 | -0.61 | -0.36*** | -0.47 | -0.25 | -0.42*** | -0.51 | -0.33 |
| BMI | -0.002 | -0.03 | 0.02 | -0.006 | -0.02 | 0.009 | 0.005 | -0.007 | 0.02 |
| Season of accelerometer wear (Ref: Summer)  *Spring* | 0.12 | -0.06 | 0.31 | 0.05 | -0.07 | 0.16 | 0.07 | -0.02 | 0.17 |
| *Autumn* | -0.03 | -0.21 | 0.15 | -0.01 | -0.13 | 0.10 | -0.01 | -0.11 | 0.08 |
| *Winter* | 0.07 | -0.13 | 0.27 | 0.02 | -0.11 | 0.15 | 0.04 | -0.06 | 0.14 |

Body mass index, BMI; Millennium Cohort Study, MCS; moderate-to-vigorous physical activity, MVPA; Strengths and Difficulties Questionnaire, SDQ

Using MCS6 accelerometer weight; interaction, ##

* p<0.05; ** p<0.01; *** p<0.001

Model 1 includes ADHD symptoms and MVPA. Model 2 adds the interaction between ADHD symptoms and MVPA over time (MVPA × wave). Model 3 further adjusts for sex, BMI, ethnicity, household income, parental education, season of accelerometer wear, and birth month.

The data in this table correspond to those displayed in Figure 1 – panels A, C, and E.

The difference in sample size between the SDQ ADHD score (n = 4,608) and the separate hyperactivity (n = 4,552) and inattention scores (n = 4,583) reflects that the total score was calculated among participants with at least three non-missing items (Goodman, Rowe & Gan, 2016), while subscales were calculated by summing items only amongst individuals who answered all items for that subscale.

Reference:

Goodman, A., Rowe, R., & Gan, Y. (2016). *Scoring the Strengths & Difficulties Questionnaire for age 4-17 or 18+*. https://www.sdqinfo.org/c3.html

Table S2. Weighted results from the mixed effects multiple linear regression models used to investigate whether the associations between ADHD symptoms and PA between females and males both at age 7 and age 14.

|  | SDQ ADHD  n=4,608 | | | Hyperactivity symptoms only  n=4,552 | | | Inattention symptoms only  n = 4,583 | | |
| --- | --- | --- | --- | --- | --- | --- | --- | --- | --- |
| Predictors | b | 95% CI | | b | 95% CI | | b | 95% CI | |
| Model 1 | | | | | | | | | |
| MVPA | 0.003 | -0.004 | 0.01 | 0.003 | -0.001 | 0.008 | 0.0003 | -0.003 | 0.003 |
| Wave (Ref: Wave 1 – MCS4)  *Wave 2 – MCS6* | -0.28 | -0.80 | -0.24 | -0.13 | -0.48 | 0.22 | -0.09 | -0.34 | 0.16 |
| Wave##MVPA (Ref: Wave 1 – MCS4)  *Wave 2 – MCS6##MVPA* | -0.0004 | -0.008 | 0.007 | -0.002 | -0.007 | 0.003 | 0.001 | -0.002 | 0.005 |
| Sex (Ref: Male)  *Female* | -1.35*** | -1.97 | -0.74 | -0.71** | -1.11 | -0.31 | -0.68*** | -0.98 | -0.38 |
| Sex##MVPA (Ref: Male)  *Female##MVPA* | 0.008 | -0.001 | 0.02 | 0.005 | -0.001 | 0.01 | 0.004 | -0.0005 | 0.008 |
| Wave##Sex (Ref: Wave 1 – MCS4, Male)  *Wave 2 – MCS6##Female* | 0.59 | -0.08 | 1.25 | 0.34 | -0.10 | 0.78 | 0.29 | -0.03 | 0.62 |
| Wave##Sex##MVPA (Ref: Wave 1 – MCS4, Male)  *Wave 2 – MCS6##Female##MVPA* | -0.008 | -0.02 | 0.002 | -0.005 | -0.01 | 0.002 | -0.004 | -0.01 | 0.0005 |
| Model 2 | | | | | | | | | |
| MVPA | 0.005 | -0.002 | 0.01 | 0.005 | -0.0002 | 0.01 | 0.001 | -0.002 | 0.004 |
| Wave (Ref: Wave 1 – MCS4)  *Wave 2 – MCS6* | -0.20 | -0.75 | 0.35 | -0.08 | -0.44 | 0.28 | -0.09 | -0.35 | 0.18 |
| Wave##MVPA (Ref Wave 1 – MCS4)  *Wave2 – MCS6##MVPA* | -0.001 | -0.008 | 0.007 | -0.002 | -0.007 | 0.003 | 0.0008 | -0.003 | 0.004 |
| Sex (Ref: Male)  *Female* | -1.23*** | -1.86 | -0.61 | -0.64** | -1.05 | -0.23 | -0.63*** | -0.94 | -0.32 |
| Sex##MVPA (Ref: Male)  *Female##MVPA* | 0.006 | -0.004 | 0.02 | 0.003 | -0.003 | 0.01 | 0.003 | -0.002 | 0.008 |
| Wave##Sex (Ref: Wave 1 – MCS4, Male)  *Wave 2 – MCS6##Female* | 0.55 | -0.12 | 1.23 | 0.36 | -0.09 | 0.80 | 0.25 | -0.08 | 0.59 |
| Wave##Sex##MVPA (Ref: Wave 1 – MCS4, Male)  *Wave 2 – MCS6##Female##MVPA* | -0.007 | -0.02 | 0.003 | -0.004 | -0.01 | 0.003 | -0.004 | -0.009 | 0.001 |
| BMI | -0.003 | -0.03 | 0.02 | -0.006 | -0.02 | 0.008 | 0.005 | -0.007 | 0.02 |
| Season of accelerometer wear (Ref: Summer)  *Spring* | 0.12 | -0.06 | 0.31 | 0.05 | -0.07 | 0.16 | 0.07 | -0.02 | 0.17 |
| *Autumn* | -0.02 | -0.20 | 0.16 | -0.007 | -0.12 | 0.11 | -0.01 | -0.10 | 0.08 |
| *Winter* | 0.07 | -0.13 | 0.27 | 0.02 | -0.11 | 0.15 | 0.04 | -0.06 | 0.14 |
| Wald Test | Chi^2^=1.83; p=0.177 | | | Chi^2^=1.49; p=0.222 | | | Chi^2^=1.92; p=0.166 | | |

Body mass index, BMI; Millennium Cohort Study, MCS; moderate-to-vigorous physical activity, MVPA; Strengths and Difficulties Questionnaire, SDQ; interaction, ##

Using MCS6 accelerometer weight.

* p<0.05; ** p<0.01; *** p<0.001

Model 1 includes ADHD symptoms and the interaction between MVPA, sex, and wave. Model 2 further adjusts for sex, BMI, ethnicity, household income, parental education, season of accelerometer wear, and birth month.

The data in this table correspond to those displayed in Figure 1 – panels B, D, and F.

###

### Appendix S4 – Predicting self-reported ADHD symptoms at age 17 using previous accelerometer-measured MVPA

Table S3. Regression models predicting self-reported SDQ ADHD score separately at age 17.

|  | | Age 17 SDQ ADHD score  Age 7, n = 2,561  Age 14, n = 2,223 | |
| --- | --- | --- | --- |
|  | | b | 95% CI |
| Combined | Age 7 MVPA | 0.004* | 0.0004 to 0.008 |
| Females | Age 7 MVPA | 0.0001 | -0.006 to 0.006 |
| Males | Age 7 MVPA | 0.006* | 0.0004 to 0.011 |
| Combined | Age 14 MVPA | 0.002 | -0.0005 to 0.005 |
| Females | Age 14 MVPA | 0.003 | -0.0006 to 0.007 |
| Males | Age 14 MVPA | 0.001 | -0.002 to 0.005 |

Attention-deficit hyperactivity disorder, ADHD; moderate-to-vigorous physical activity, MVPA Strengths and Difficulties Questionnaire, SDQ

Using MCS4 accelerometer weight for age 7, using MCS6 accelerometer weight for age 14.

Models adjusted for age, birth month, ethnicity, parental income and parental highest level of academic qualification.

* p<0.05; ** p<0.01; *** p<0.001

Note: No p-values (two-tailed) were significant after multiple testing correction (5% false discovery rate [FDR]).

### Appendix S5 – Associations between reported ADHD symptoms and reported physical activity

Table S4. Weighted results from the multiple linear regression analyses regarding age 7 (MCS4) SDQ ADHD, hyperactivity symptoms only and inattention symptoms only using parent-reported subjective physical activity levels: regression coefficients and 95% CIs.

|  | Combined  n = 13,215 | | | Females  n = 6,515 | | | Males  n = 6,700 | | |
| --- | --- | --- | --- | --- | --- | --- | --- | --- | --- |
| Predictors | b | 95% CI | | b | 95% CI | | b | 95% CI | |
| SDQ ADHD score | | | | | | | | | |
| Days per week CM does sport/exercise (Ref: <once a week) |  |  |  |  |  |  |  |  |  |
| *One day a week* | -0.23** | -0.36 | -0.09 | -0.20* | -0.39 | -0.01 | -0.25** | -0.42 | -0.07 |
| *Two days a week* | -0.39*** | -0.53 | -0.26 | -0.34** | -0.54 | -0.14 | -0.45*** | -0.63 | -0.27 |
| *Three days a week* | -0.42*** | -0.58 | -0.26 | -0.31** | -0.52 | -0.10 | -0.52*** | -0.74 | -0.29 |
| *Four or more days a week* | -0.40*** | -0.60 | -0.20 | -0.51*** | -0.79 | -0.23 | -0.31* | -0.59 | -0.03 |
| Sex (Ref: Male)  *Female* | -0.90*** | -1.00 | -0.80 |  |  |  |  |  |  |
| BMI | 0.004 | -0.02 | 0.03 | -0.007 | -0.03 | 0.02 | 0.02 | -0.01 | 0.05 |
| Hyperactivity symptoms only | | | | | | | | | |
| Days per week CM does sport/exercise (Ref: <once a week) |  |  |  |  |  |  |  |  |  |
| *One day a week* | -0.16*** | -0.25 | -0.07 | -0.13* | -0.25 | -0.007 | -0.19** | -0.30 | -0.07 |
| *Two days a week* | -0.24*** | -0.33 | -0.14 | -0.18** | -0.31 | -0.05 | -0.30*** | -0.43 | -0.17 |
| *Three days a week* | -0.23*** | -0.34 | -0.13 | -0.15* | -0.28 | -0.008 | -0.33*** | -0.48 | -0.18 |
| *Four or more days a week* | -0.21** | -0.33 | -0.08 | -0.22* | -0.39 | -0.05 | -0.20* | -0.37 | -0.02 |
| Sex (Ref: Male)  *Female* | -0.50*** | -0.56 | -0.44 |  |  |  |  |  |  |
| BMI | -0.003 | -0.02 | 0.01 | -0.009 | -0.03 | 0.008 | 0.04 | -0.02 | 0.02 |
| Inattention symptoms only | | | | | | | | | |
| Days per week CM does sport/exercise (Ref: <once a week) |  |  |  |  |  |  |  |  |  |
| *One day a week* | -0.07 | -0.13 | 0.0007 | -0.06 | -0.15 | 0.03 | -0.07 | -0.16 | 0.02 |
| *Two days a week* | -0.15*** | -0.22 | -0.09 | -0.16** | -0.26 | -0.06 | -0.15** | -0.23 | -0.06 |
| *Three days a week* | -0.17*** | -0.25 | -0.09 | -0.15** | -0.25 | -0.04 | -0.19** | -0.30 | -0.07 |
| *Four or more days a week* | -0.19*** | -0.29 | -0.09 | -0.29*** | -0.43 | -0.14 | -0.11 | -0.25 | 0.02 |
| Sex (Ref: Male)  *Female* | -0.42*** | -0.47 | -0.37 |  |  |  |  |  |  |
| BMI | 0.006 | -0.004 | 0.02 | 0.003 | -0.01 | 0.02 | 0.01 | -0.25 | 0.02 |

Body mass index, BMI; cohort member, CM; Strengths and Difficulties Questionnaire, SDQ

Using MCS4 overall sampling weight for age 7.

Models adjusted for age, birth month, ethnicity, parental income and parental highest level of academic qualification.

* p<0.05; ** p<0.01; *** p<0.001

Note: All p-values remained significant after multiple testing correction (5% false discovery rate [FDR]).

Table S5. Weighted results from the multiple linear regression analyses regarding age 11 (MCS5) SDQ ADHD, hyperactivity symptoms only and inattention symptoms only using parent-reported subjective physical activity levels: regression coefficients and 95% CIs.

|  | Combined  n = 12,301 | | | Females  n = 6,082 | | | Males  n = 6,219 | | |
| --- | --- | --- | --- | --- | --- | --- | --- | --- | --- |
| Predictors | b | 95% CI | | b | 95% CI | | b | 95% CI | |
| SDQ ADHD score | | | | | | | | | |
| Days per week CM does sport/exercise (Ref: <once a week) |  |  |  |  |  |  |  |  |  |
| *One day a week* | -0.16 | -0.33 | 0.007 | -0.17 | -0.36 | 0.03 | -0.17 | -0.43 | 0.09 |
| *Two days a week* | -0.38*** | -0.54 | -0.22 | -0.33** | -0.51 | -0.14 | -0.44** | -0.71 | -0.16 |
| *Three days a week* | -0.52*** | -0.68 | -0.36 | -0.46*** | -0.66 | -0.26 | -0.59*** | -0.84 | -0.33 |
| *Four or more days a week* | -0.57*** | -0.74 | -0.39 | -0.33** | -0.55 | -0.11 | -0.77*** | -1.02 | -0.52 |
| Sex (Ref: Male)  *Female* | -1.01*** | -1.11 | -0.91 |  |  |  |  |  |  |
| BMI | 0.007 | -0.008 | 0.02 | 0.02* | 0.001 | 0.04 | -0.005 | -0.03 | 0.02 |
| Hyperactivity symptoms only | | | | | | | | | |
| Days per week CM does sport/exercise (Ref: <once a week) |  |  |  |  |  |  |  |  |  |
| *One day a week* | -0.09 | -0.20 | 0.01 | -0.06 | -0.19 | 0.06 | -0.14 | -0.31 | 0.03 |
| *Two days a week* | -0.21*** | -0.32 | -0.10 | -0.18** | -0.30 | -0.06 | -0.25** | -0.43 | -0.07 |
| *Three days a week* | -0.26*** | -0.36 | -0.17 | -0.18** | -0.31 | -0.06 | -0.35*** | -0.50 | -0.19 |
| *Four or more days a week* | -0.25*** | -0.36 | -0.14 | -0.14 | -0.27 | 0.03 | -0.37*** | -0.53 | -0.20 |
| Sex (Ref: Male)  *Female* | -0.54*** | -0.61 | -0.48 |  |  |  |  |  |  |
| BMI | 0.002 | -0.008 | 0.01 | 0.01 | -0.0007 | 0.02 | -0.006 | -0.02 | 0.01 |
| Inattention symptoms only | | | | | | | | | |
| Days per week CM does sport/exercise (Ref: <once a week) |  |  |  |  |  |  |  |  |  |
| *One day a week* | -0.09* | -0.17 | -0.007 | -0.14** | -0.24 | -0.04 | -0.04 | -0.16 | 0.09 |
| *Two days a week* | -0.17*** | -0.25 | -0.10 | -0.15** | -0.24 | -0.05 | -0.19** | -0.31 | -0.06 |
| *Three days a week* | -0.27*** | -0.36 | -0.18 | -0.29*** | -0.40 | -0.17 | -0.26*** | -0.39 | -0.13 |
| *Four or more days a week* | -0.32*** | -0.40 | -0.23 | -0.21*** | -0.32 | -0.09 | -0.40*** | -0.51 | -0.28 |
| Sex (Ref: Male)  *Female* | -0.47*** | -0.52 | -0.42 |  |  |  |  |  |  |
| BMI | 0.006 | -0.0006 | 0.01 | 0.009* | 0.0002 | 0.02 | 0.002 | -0.008 | 0.01 |

Body mass index, BMI; cohort member, CM; Strengths and Difficulties Questionnaire, SDQ

Using MCS5 overall sampling weight for age 11.

Models adjusted for age, birth month, ethnicity, parental income and parental highest level of academic qualification.

* p<0.05; ** p<0.01; *** p<0.001

Note: All p-values remained significant after multiple testing correction (5% false discovery rate [FDR]).

Table S6. Weighted results from the multiple linear regression analyses regarding age 11 (MCS5) SDQ ADHD, hyperactivity symptoms only and inattention symptoms only using self-reported subjective physical activity levels: regression coefficients and 95% CIs.

|  | Combined  n = 12,054 | | | Females  n = 5,984 | | | Males  n = 6,070 | | |
| --- | --- | --- | --- | --- | --- | --- | --- | --- | --- |
| Predictors | b | 95% CI | | b | 95% CI | | b | 95% CI | |
| SDQ ADHD score | | | | | | | | | |
| How often CM plays sport or active games (Ref: never) |  |  |  |  |  |  |  |  |  |
| *Less often than once a month* | -0.71** | -1.19 | -0.23 | -0.87** | -1.48 | -0.26 | -0.57 | -1.27 | 0.14 |
| *At least once a month* | -0.99*** | -1.40 | -0.59 | -1.13*** | -1.61 | -0.65 | -0.87* | -1.54 | -0.20 |
| *At least once a week* | -1.01*** | -1.36 | -0.65 | -1.12*** | -1.56 | -0.68 | -0.91** | -1.48 | -0.34 |
| *Most days* | -1.08*** | -1.43 | -0.72 | -1.11*** | -1.54 | -0.68 | -1.06*** | -1.63 | -0.50 |
| Sex (Ref: Male)  *Female* | -0.98*** | -1.08 | -0.88 |  |  |  |  |  |  |
| BMI | 0.007 | -0.008 | 0.02 | 0.02* | 0.00001 | 0.04 | -0.005 | -0.03 | 0.02 |
| Hyperactivity symptoms only | | | | | | | | | |
| How often CM plays sport or active games (Ref: never) |  |  |  |  |  |  |  |  |  |
| *Less often than once a month* | -0.47** | -0.77 | -0.18 | -0.58** | -0.96 | -0.21 | -0.38 | -0.83 | 0.07 |
| *At least once a month* | -0.66*** | -0.91 | -0.42 | -0.71*** | -1.01 | -0.42 | -0.64** | -1.06 | -0.22 |
| *At least once a week* | -0.60*** | -0.82 | -0.37 | -0.65*** | -0.92 | -0.38 | -0.56** | -0.94 | -0.19 |
| *Most days* | -0.63*** | -0.84 | -0.41 | -0.65*** | -0.92 | -0.39 | -0.62** | -0.98 | -0.27 |
| Sex (Ref: Male)  *Female* | -0.53*** | -0.59 | -0.46 |  |  |  |  |  |  |
| BMI | 0.002 | -0.009 | 0.01 | 0.009 | -0.003 | 0.02 | -0.005 | -0.02 | 0.01 |
| Inattention symptoms only | | | | | | | | | |
| How often CM plays sport or active games (Ref: never) |  |  |  |  |  |  |  |  |  |
| *Less often than once a month* | -0.29* | -0.53 | -0.05 | -0.30 | -0.62 | 0.03 | -0.30 | -0.61 | 0.01 |
| *At least once a month* | -0.36*** | -0.57 | -0.16 | -0.41** | -0.67 | -0.15 | -0.31 | -0.63 | 0.02 |
| *At least once a week* | -0.47*** | -0.64 | -0.29 | -0.48*** | -0.72 | -0.24 | -0.46*** | -0.71 | -0.21 |
| *Most days* | -0.51*** | -0.69 | -0.34 | -0.47*** | -0.71 | -0.24 | -0.56*** | -0.81 | -0.31 |
| Sex (Ref: Male)  *Female* | -0.46*** | -0.51 | -0.41 |  |  |  |  |  |  |
| BMI | 0.006 | -0.001 | 0.01 | 0.01* | 0.0001 | 0.02 | 0.001 | -0.009 | 0.01 |

Body mass index, BMI; cohort member, CM; Strengths and Difficulties Questionnaire, SDQ

Using MCS5 overall sampling weight for age 11.

Models adjusted for age, birth month, ethnicity, parental income and parental highest level of academic qualification.

* p<0.05; ** p<0.01; *** p<0.001

Note: All p-values remained significant after multiple testing correction (5% false discovery rate [FDR]).

Table S7. Weighted results from the multiple linear regression analyses regarding age 14 (MCS6) SDQ ADHD, hyperactivity symptoms only and inattention symptoms only using self-reported subjective physical activity levels: regression coefficients and 95% CIs.

|  | Combined  n = 10,439 | | | Females  n = 5,143 | | | Males  n = 5,296 | | |
| --- | --- | --- | --- | --- | --- | --- | --- | --- | --- |
| Predictors | b | 95% CI | | b | 95% CI | | b | 95% CI | |
| SDQ ADHD | | | | | | | | | |
| Days last week spent doing MVPA (Ref: Not at all) |  |  |  |  |  |  |  |  |  |
| *1-2 days per week* | -0.62*** | -0.94 | -0.30 | -0.69** | -1.12 | -0.25 | -0.55* | -0.98 | -0.11 |
| *3-4 days per week* | -0.77*** | -1.07 | -0.46 | -0.80*** | -1.22 | -0.38 | -0.75** | -1.19 | -0.30 |
| *5-6 days per week* | -0.81*** | -1.14 | -0.49 | -0.82*** | -1.28 | -0.37 | -0.81** | -1.26 | -0.35 |
| *Every day* | -0.43* | -0.75 | -0.10 | -0.49* | -0.95 | -0.02 | -0.40 | -0.84 | 0.05 |
| Sex (Ref: Male)  *Female* | -0.90*** | -1.03 | -0.77 |  |  |  |  |  |  |
| BMI | 0.02* | 0.0006 | 0.03 | 0.02 | -0.003 | 0.03 | 0.02 | -0.006 | 0.04 |
| Hyperactivity symptoms only | | | | | | | | | |
| Days last week spent doing MVPA (Ref: Not at all) |  |  |  |  |  |  |  |  |  |
| *1-2 days per week* | -0.30** | -0.50 | -0.11 | -0.28* | -0.53 | -0.04 | -0.33* | -0.62 | -0.04 |
| *3-4 days per week* | -0.36*** | -0.54 | -0.18 | -0.30* | -0.54 | -0.06 | -0.44** | -0.73 | -0.14 |
| *5-6 days per week* | -0.33*** | -0.53 | -0.13 | -0.26* | -0.53 | -0.001 | -0.41** | -0.72 | -0.10 |
| *Every day* | -0.09 | -0.29 | 0.11 | -0.05 | -0.33 | 0.23 | -0.15 | -0.45 | 0.14 |
| Sex (Ref: Male)  *Female* | -0.43*** | -0.51 | -0.36 |  |  |  |  |  |  |
| BMI | 0.007 | -0.003 | 0.02 | 0.006 | -0.005 | 0.02 | 0.007 | -0.007 | 0.02 |
| Inattention symptoms only | | | | | | | | | |
| Days last week spent doing MVPA (Ref: Not at all) |  |  |  |  |  |  |  |  |  |
| *1-2 days per week* | -0.30*** | -0.46 | -0.13 | -0.35** | -0.59 | -0.12 | -0.23* | -0.45 | -0.01 |
| *3-4 days per week* | -0.39*** | -0.55 | -0.23 | -0.46*** | -0.69 | -0.23 | -0.33** | -0.55 | -0.10 |
| *5-6 days per week* | -0.46*** | -0.63 | -0.30 | -0.51*** | -0.76 | -0.26 | -0.41*** | -0.63 | -0.19 |
| *Every day* | -0.31*** | -0.47 | -0.14 | -0.37** | -0.62 | -0.13 | -0.24* | -0.46 | -0.03 |
| Sex (Ref: Male)  *Female* | -0.36*** | -0.53 | -0.39 |  |  |  |  |  |  |
| BMI | 0.008* | 0.0006 | 0.02 | 0.007 | -0.002 | 0.02 | 0.009 | -0.002 | 0.02 |

Body mass index, BMI; cohort member, CM; moderate-to-vigorous physical activity, MVPA; Strengths and Difficulties Questionnaire, SDQ

Using MCS6 overall sampling weight for age 14.

Models adjusted for age, birth month, ethnicity, parental income and parental highest level of academic qualification.

* p<0.05; ** p<0.01; *** p<0.001 Note: All p-values remained significant after multiple testing correction (5% false discovery rate [FDR]).

### Appendix S6 – Benjamini Hochberg corrections

Below are the input values, calculations and resulting Benjamini and Hochberg corrected significance level for the 3 central research questions. The Benjamini and Hochberg value means that, when your intended level of significance corresponds to the value of q entered in the DataInput table, all p-values below the provided value of q* show significant results. The correction after Benjamini and Hochberg (1995) is dependent on the calculated p-values of your tests.

**Table S8. Research question 1**

Input values

| *p*-values | Level of significance | |
| --- | --- | --- |
| 0.001 | *q* = | 0.050 |
| 0.001 |  |  |
| 0.001 |  |  |
| 0.001 |  |  |
| 0.001 |  |  |
| 0.001 |  |  |
| 0.093 |  |  |
| 0.069 |  |  |
| 0.328 |  |  |
| 0.001 |  |  |
| 0.075 |  |  |
| 0.003 |  |  |
| 0.003 |  |  |
| 0.085 |  |  |
| 0.006 |  |  |
| 0.015 |  |  |
| 0.258 |  |  |
| 0.015 |  |  |

| *Calculations and results* | | | | | |
| --- | --- | --- | --- | --- | --- |
| ***i*** | ***p*** | | ***q**** | ***p* < *q**** | **Find** |
| 1 | 0.0010 | | 0.0028 | TRUE | -1 |
| 2 | 0.0010 | | 0.0056 | TRUE | -1 |
| 3 | 0.0010 | | 0.0083 | TRUE | -1 |
| 4 | 0.0010 | | 0.0111 | TRUE | -1 |
| 5 | 0.0010 | | 0.0139 | TRUE | -1 |
| 6 | 0.0010 | | 0.0167 | TRUE | -1 |
| 7 | 0.0010 | | 0.0194 | TRUE | -1 |
| 8 | 0.0030 | | 0.0222 | TRUE | -1 |
| 9 | 0.0030 | | 0.0250 | TRUE | -1 |
| 10 | 0.0060 | | 0.0278 | TRUE | -1 |
| 11 | 0.0150 | | 0.0306 | TRUE | -1 |
| 12 | 0.0150 | | 0.0333 | TRUE | 12 |
| 13 | 0.0690 | | 0.0361 | FALSE | 1000 |
| 14 | 0.0750 | | 0.0389 | FALSE | 1000 |
| 15 | 0.0850 | | 0.0417 | FALSE | 1000 |
| 16 | 0.0930 | | 0.0444 | FALSE | 1000 |
| 17 | 0.2580 | | 0.0472 | FALSE | 1000 |
| 18 | 0.3280 | | 0.0500 | FALSE | 1000 |
| Benjamini and Hochberg (1995) corrected significance level | | | | | |
| *q** = | | 0.033333333 | | |  |

**Table S9. Research question 2**

Input values

| *p*-values | Level of significance | |
| --- | --- | --- |
| 0.003 | *q* = | 0.050 |
| 0.389 |  |  |
| 0.007 |  |  |
| 0.063 |  |  |
| 0.652 |  |  |
| 0.014 |  |  |
| 0.843 |  |  |
| 0.23 |  |  |
| 0.037 |  |  |
| 0.031 |  |  |
| 0.976 |  |  |
| 0.037 |  |  |
| 0.515 |  |  |
| 0.754 |  |  |
| 0.449 |  |  |
| 0.226 |  |  |
| 0.076 |  |  |
| 0.6 |  |  |

Calculations and results

|  |  |  |  |  |
| --- | --- | --- | --- | --- |
| ***i*** | ***p*** | ***q**** | ***p* < *q**** | **Find** |
| 1 | 0.0030 | 0.0028 | FALSE | 1000 |
| 2 | 0.0070 | 0.0056 | FALSE | 1000 |
| 3 | 0.0140 | 0.0083 | FALSE | 1000 |
| 4 | 0.0310 | 0.0111 | FALSE | 1000 |
| 5 | 0.0370 | 0.0139 | FALSE | 1000 |
| 6 | 0.0370 | 0.0167 | FALSE | 1000 |
| 7 | 0.0630 | 0.0194 | FALSE | 1000 |
| 8 | 0.0760 | 0.0222 | FALSE | 1000 |
| 9 | 0.2260 | 0.0250 | FALSE | 1000 |
| 10 | 0.2300 | 0.0278 | FALSE | 1000 |
| 11 | 0.3890 | 0.0306 | FALSE | 1000 |
| 12 | 0.4490 | 0.0333 | FALSE | 1000 |
| 13 | 0.5150 | 0.0361 | FALSE | 1000 |
| 14 | 0.6000 | 0.0389 | FALSE | 1000 |
| 15 | 0.6520 | 0.0417 | FALSE | 1000 |
| 16 | 0.7540 | 0.0444 | FALSE | 1000 |
| 17 | 0.8430 | 0.0472 | FALSE | 1000 |
| 18 | 0.9760 | 0.0500 | FALSE | 1000 |
| Benjamini and Hochberg (1995) corrected significance level | | | | |
| *q** = | None of your multiple tests yielded significant results | | |  |

**Table S10. Research question 3**

Input values

| *p*-values | Level of significance | |
| --- | --- | --- |
| 0.001 | *q* = | 0.050 |
| 0.001 |  |  |
| 0.001 |  |  |
| 0.001 |  |  |
| 0.036 |  |  |
| 0.001 |  |  |
| 0.001 |  |  |
| 0.0004 |  |  |
| 0.0036 |  |  |
| 0.001 |  |  |
| 0.001 |  |  |
| 0.001 |  |  |
| 0.001 |  |  |
| 0.0118 |  |  |
| 0.001 |  |  |
| 0.001 |  |  |
| 0.001 |  |  |
| 0.001 |  |  |
| 0.001 |  |  |
| 0.001 |  |  |
| 0.001 |  |  |
| 0.001 |  |  |
| 0.001 |  |  |
| 0.0096 |  |  |
| 0.001 |  |  |
| 0.0007 |  |  |
| 0.001 |  |  |
| 0.001 |  |  |
| 0.0019 |  |  |
| 0.0013 |  |  |
| 0.001 |  |  |
| 0.0085 |  |  |
| 0.0006 |  |  |
| 0.001 |  |  |
| 0.0003 |  |  |
| 0.041 |  |  |

Calculations and results

| ***i*** | ***p*** | ***q**** | ***p* < *q**** | **Find** |
| --- | --- | --- | --- | --- |
| 1 | 0.0003 | 0.0014 | TRUE | -1 |
| 2 | 0.0004 | 0.0028 | TRUE | -1 |
| 3 | 0.0006 | 0.0042 | TRUE | -1 |
| 4 | 0.0007 | 0.0056 | TRUE | -1 |
| 5 | 0.0010 | 0.0069 | TRUE | -1 |
| 6 | 0.0010 | 0.0083 | TRUE | -1 |
| 7 | 0.0010 | 0.0097 | TRUE | -1 |
| 8 | 0.0010 | 0.0111 | TRUE | -1 |
| 9 | 0.0010 | 0.0125 | TRUE | -1 |
| 10 | 0.0010 | 0.0139 | TRUE | -1 |
| 11 | 0.0010 | 0.0153 | TRUE | -1 |
| 12 | 0.0010 | 0.0167 | TRUE | -1 |
| 13 | 0.0010 | 0.0181 | TRUE | -1 |
| 14 | 0.0010 | 0.0194 | TRUE | -1 |
| 15 | 0.0010 | 0.0208 | TRUE | -1 |
| 16 | 0.0010 | 0.0222 | TRUE | -1 |
| 17 | 0.0010 | 0.0236 | TRUE | -1 |
| 18 | 0.0010 | 0.0250 | TRUE | -1 |
| 19 | 0.0010 | 0.0264 | TRUE | -1 |
| 20 | 0.0010 | 0.0278 | TRUE | -1 |
| 21 | 0.0010 | 0.0292 | TRUE | -1 |
| 22 | 0.0010 | 0.0306 | TRUE | -1 |
| 23 | 0.0010 | 0.0319 | TRUE | -1 |
| 24 | 0.0010 | 0.0333 | TRUE | -1 |
| 25 | 0.0010 | 0.0347 | TRUE | -1 |
| 26 | 0.0010 | 0.0361 | TRUE | -1 |
| 27 | 0.0010 | 0.0375 | TRUE | -1 |
| 28 | 0.0010 | 0.0389 | TRUE | -1 |
| 29 | 0.0013 | 0.0403 | TRUE | -1 |
| 30 | 0.0019 | 0.0417 | TRUE | -1 |
| 31 | 0.0036 | 0.0431 | TRUE | -1 |
| 32 | 0.0085 | 0.0444 | TRUE | -1 |
| 33 | 0.0096 | 0.0458 | TRUE | -1 |
| 34 | 0.0118 | 0.0472 | TRUE | -1 |
| 35 | 0.0360 | 0.0486 | TRUE | -1 |
| 36 | 0.0410 | 0.0500 | TRUE | 36 |
| Benjamini and Hochberg (1995) corrected significance level | | | | |
| *q** =0.05 | | | | |
